# Research progress of nanomaterial drug delivery in tumor targeted therapy

**DOI:** 10.3389/fbioe.2023.1240529

**Published:** 2023-07-24

**Authors:** Peng Zhang, Guihua Ye, Guofeng Xie, Jie Lv, Xianhai Zeng, Wei Jiang

**Affiliations:** ^1^ Department of Otorhinolaryngology, Longgang Otorhinolaryngology Hospital & Shenzhen Key Laboratory of Otorhinolaryngology, Shenzhen Institute of Otorhinolaryngology, Shenzhen, China; ^2^ Shanghai Ninth People’s Hospital Hainan Branch, Hainan Western Central Hospital, Danzhou, China; ^3^ Affiliated Cancer Hospital and Institute of Guangzhou Medical University, Guangzhou, China; ^4^ School of Computer Science and Engineering, Yulin Normal University, Yulin, China

**Keywords:** nanomaterials, tumor, targeted therapy, drug delivery, tumorigenic factors

## Abstract

Cancer is one of the most lethal diseases in human society, and its incidence is gradually increasing. However, the current tumor treatment often meets the problem of poor efficacy and big side effects. The unique physical and chemical properties of nanomaterials can target the delivery of drugs to tumors, which can improve the therapeutic effect while reducing the damage of drugs to normal cells. This makes nanomaterials become a hot topic in the field of biomedicine. This review summarizes the recent progress of nanomaterials in tumor targeted therapy.

## 1 Introduction

Malignant tumor is a new organism, that is seriously disturbed in the regulation of cell growth under the action of various tumorigenic factors, resulting in the abnormal proliferation of cells in the body. It often presents as abnormal tissue clumps in the body. Tumors can escape the surveillance of the immune system, grow without limit, and metastasize through blood, lymphatic, or implantation (Robert, 2013). There are still great challenges in the treatment of malignant tumors. In 2022, 1918030 new cancer cases and 609360 cancer deaths are projected to occur in the United States. Although the incidence of lung cancer is gradually slowing down, the incidence of breast cancer and advanced stage prostate cancer is still increasing (Siegel and Miller, 2022). According to the National Cancer Center of China, the number of new cases of malignant tumor in China in 2016 was 4.0640 million, of which 2.2343 million were men and 1.829,600 were women. The crude and age-standardized incidence rates (ASIR) were 293.91 and 186.46 per 100,000 population, respectively (Zheng et al., 2022). Nowadays, the traditional treatment methods are mainly surgery, chemotherapy and radiotherapy. However, the therapeutic effect is still not satisfactory. The main reasons include side effects, drug resistance and insensitivity of tumor cells to radiation (Zhang et al., 2018). Therefore, the search for a highly targeted, efficient and low toxicity therapy has become an important direction in cancer therapy research.

At present, many targeted drugs have been applied in clinical practice, which have the advantages of good targeting and few side effects. Targeted therapy can inhibit the growth of tumor cells by targeting drugs on key genes of tumor growth and division, such as EGFR, HER-2, KRAS, ALK, etc (Rouviere et al., 2015; Saito et al., 2018; Meric-Bernstam et al., 2019). However, the mutation rate of target genes in patient population is not high, so many patients are not suitable for using targeted drugs. In addition, in clinical practice, targeted drugs may miss the target. Long-term use is easy to resist drugs, and the price is expensive. Therefore, it has broad application prospect to find a new therapeutic method which is applicable to a wide population and stable targeting performance. At present, the commonly used targeted drugs in clinical practice include Bevacizumab, Trastuzumab, Cetuximab, Pertuzumab, Osimertinib, Lenvatinib, etc. These targeted drugs can mainly be used in the treatment of lung cancer, bowel cancer, breast cancer, liver cancer, lymphoma.

**FIGURE 1:**
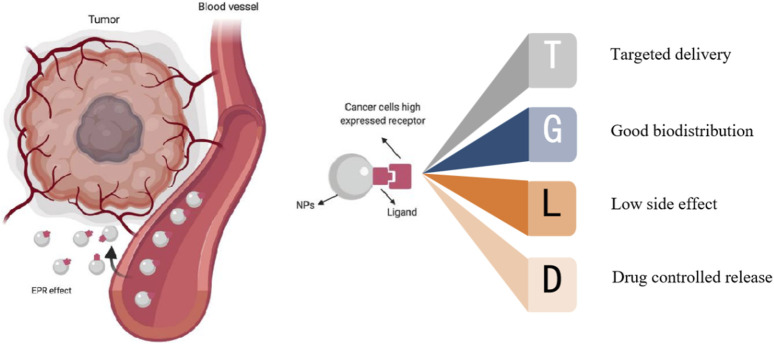
Targeting of nanoparticles: active targeting and passive targeting (Sanità et al., 2020).

Nanoparticles exhibit unique physical, chemical, and biological properties. They find wide-ranging applications in fields such as biomedical research (Schmalz et al., 2017). The characteristics of small size and large area to volume ratio of nanoparticles enable them to efficiently bind, absorb and deliver small molecules of drugs (Yetisgin and Cetinel, 2020). Their variable size, shape and surface characteristics also enable them to have high stability, high carrying capacity, properties of binding hydrophilic or hydrophobic substances, and compatibility of different drug delivery routes. Because of their own advantages, nanomaterials can play a certain advantage in tumor treatment. Nanomaterials can wrap drugs or combine drugs to be targeted and delivered near the tumor area, so that drugs can act on tumor cells more accurately. The targeted therapy of nanomaterials can be divided into passive and active targeting according to different targeting pathways (Alavi and Hamidi, 2019). The former refers to that after the combination of nanomaterials and therapeutic drugs enters the human body, taking into account the differences in the specific biochemical microenvironment of organs, tissues, cells and specific lesions, the drugs will stop, stay or accumulate in special sites, so as to increase the efficacy and concentration of drugs at the tumor site, so as to achieve targeted treatment. The latter uses artificial means to deliver nanomaterials and drugs near the tumor site for selective treatment (Figure 1).

There is a difference between targeted drugs and nanomaterial targeted therapy, targeted drugs are targeted to the tumor site through the specific binding of drug molecules to specific receptors on the surface of tumor cells, while nanomaterial is targeted to the tumor site through the unique physical and chemical properties of nanomaterials, such as magnetic properties, EPR effects, and binding tumor recognition ligands. Compared with direct intravenous drug input, encapsulation or combination of nanomaterials has the following advantages (Hou et al., 2020; Barani et al., 2021; Dutta et al., 2021; Kashyap et al., 2023): 1) It can improve drug targeting for tumor and reduce the damage to normal tissues and organs. 2) It can improve drug stability, reduce drug delivery by enzymatic hydrolysis, resulting in drug destruction. It can reduce the side effect of anticancer agents. 3) It can make the drug in the body controlled release. For example, the prodrug nanoassemblies (LPNAs) and the pH-reduction dual responsive drug delivery system designed by Ding et al. (2019) can precisely control drug release, resulting in better therapeutic effects (Ding et al., 2019; Ding et al., 2023). In addition, while delivering drugs, nanomaterials can also produce photothermal effects and enhance the killing effect on tumors. Based on the numerous advantages of nanomaterials, nanomaterials have been widely used in the drug-loaded targeted therapy of cancer. This paper will review the research progress in this direction, and provide certain reference value for the subsequent research.

## 2 Commonly used nanomaterials

**FIGURE 2:**
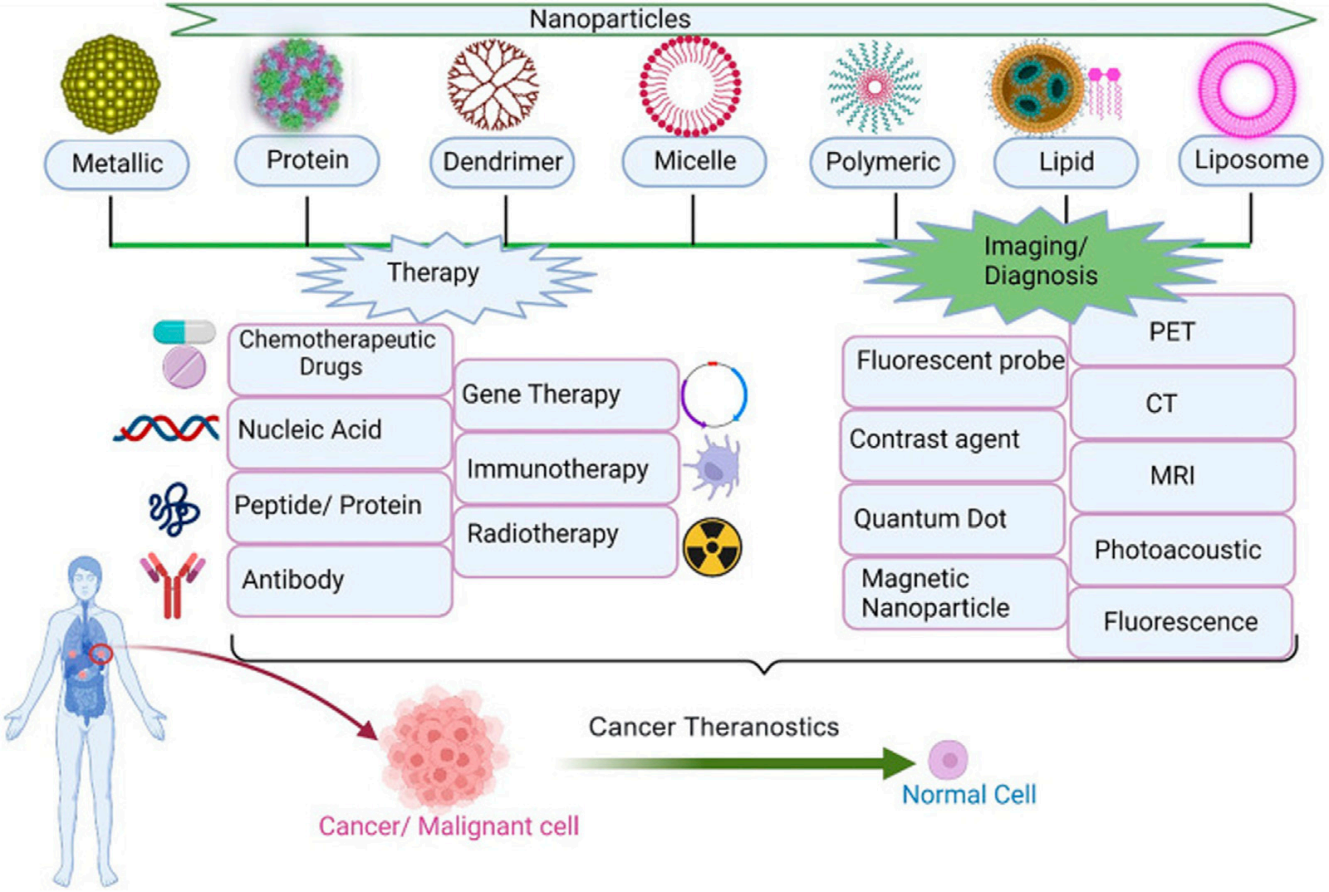
Application of nanoparticles in tumor therapy and diagnosis (Kashyap et al., 2023).

Due to the numerous advantages, nanomaterials have been wildly used in clinical and laboratory researches (Figure 2). In this section, we summary the commonly used nanomaterials in cancer therapy, including organic nanomaterials and inorganic nanomaterials (Khalid et al., 2020). At present, some nano-drugs have been applied in clinical practice. For example, Abraxane and Paclitaxel liposome are commonly used in the treatment of breast cancer, ovarian cancer, and non-small cell lung cancer. Adriamycin liposomes is often used in the treatment of ovarian cancer, non-Hodgkin lymphoma, breast cancer, and uterine tumors.

### 2.1 Organic nanomaterials

Organic nanomaterials are substances that mostly naturally existing in organisms or synthesized by chemicals. Such nanomaterials are less cytotoxic and biodegradable, so they are currently a research hotspot and widely used (Khizar et al., 2023). Organic nanomaterials have the advantages of controllable size, large specific surface area, easy surface modification, high permeability and retention effect, and good biocompatibility (López-Dávila et al., 2012; Hussein Kamareddine et al., 2019). Organic nanomaterials can carry both hydrophilic and hydrophobic drugs. Modification of endogenous molecules of some receptors on the surface of organic nanomaterials can significantly improve the targeting of tumor tissue. At present, organic nanomaterials mainly include liposome, protein and polymeric nanomaterials (Palazzolo et al., 2018; Wang et al., 2021).

#### 2.1.1 Liposome nanomaterials

Liposomes are nanocapsules with closed spherical vesicle structure surrounded by phospholipid bilayer and contain hydrophilic water nuclei, which can bind both hydrophilic and hydrophobic drugs (Yang et al., 2021). Compared with other nanocarriers, liposome show good biocompatibility and high drug loading capacity, showing lower toxicity, low immunogenicity, and can be cleared through normal metabolism.

**FIGURE 3:**
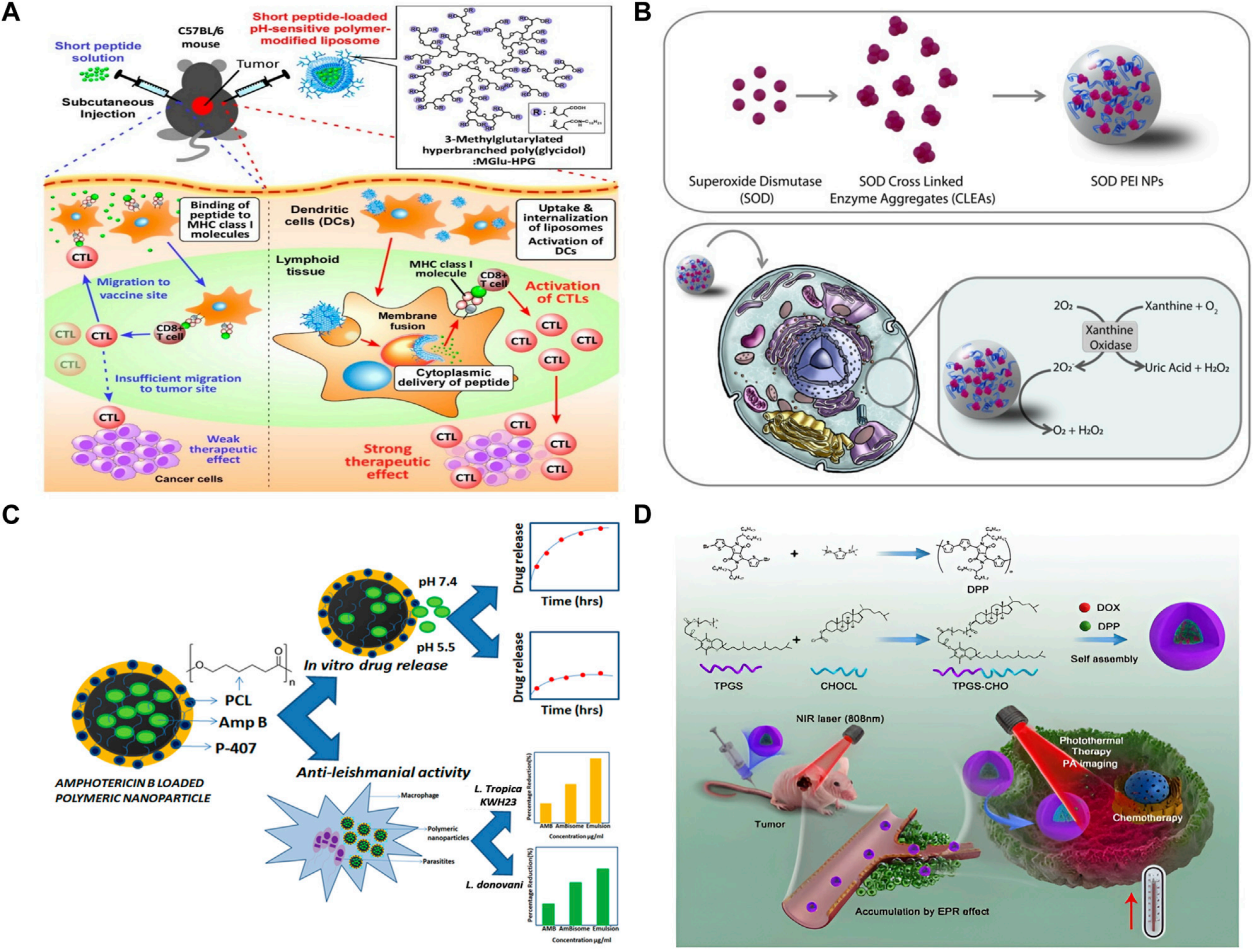
Organic nanomaterial. **(A)** Antigen delivery of pH-sensitive polymer-modified liposomes (Yoshizaki et al., 2016). **(B)** The synthesis, *in vitro* assay, and activity in cell of SOD NPs (Galliani et al., 2019). **(C)** From Amphotericin B (Amp B) loaded polymeric nanoparticles preparation to *in vitro* drug release (Saqib et al., 2020). **(D)** Polymer-Based nanocarriers for Co-Delivery and combination of diverse therapies against cancers (Yan et al., 2018).

At present, the clinical application of liposome preparations contain different chemotherapy drugs, such as Doxil1/Caelyx1/Myocet1, DaunoXome1, and DepoCyte1, respectively, were used to treat patients with ovarian cancer, AIDS-associated Kaposi’s sarcoma, multiple myeloma, lymphoma, or leukemia combined with meningeal diffusion (Kaspers et al., 2013; Bhowmik et al., 2018; Le Rhun et al., 2020). In addition, there are a number of drugs are being studied. Anthracyclines (ANT) are aromatic polyketo antitumor drugs, representative of which are doxorubicin (DOX), daunorubicin (DNR), aclarubicin, epirubicin, pirarubicin, etc. Doxil is FDA approved for the treatment of ovarian cancer and Kaposi’s sarcoma (Addeo et al., 2008). One of the most common dose-limiting toxicities of anthracyclines is cardiotoxicity, especially in the elderly and in patients with severe complications such as diabetes and coronary heart disease. However, cardiotoxic events with anthracyclines encapsulated in liposomes are significantly reduced during tumor therapy (Fassas et al., 2002). As is shown in Figure 3A, Yoshizaki et al. (2016) a pH-sensitive fusogenic polymer-modified liposomes. The liposomes were loaded with antigenic peptides derived from ovalbumin (OVA) OVA-I (SIINFEKL), and OVA-II, which can significantly enhance the activation efficiency of cytotoxic T lymphocyte (ctl), so as to achieve efficient cancer immunotherapy. Matbou Riahi et al. (2018) developed celecoxib (CLX) liposome nanomaterials to overcome the shortcomings of poor water solubility and low anti-tumor titer, and found that CLX liposome preparation had the slowest release curve and the strongest anti-tumor effect *in vivo*. Compared with free CLX, the accumulation of CLX liposomes in tumor sites increased by 3 times. It is proved that CLX liposome is a safe and effective antitumor agent and can release the drug slowly. Wang S. et al. (2020b) used the copper acetate gradient method to prepare As_2_O_3_ liposomes. By forming a complex of aqueous copper acetate and As_2_O_3_ in the liposomes, the encapsulation rate reached 83.1%, the tumor inhibition rate reached 61.2%, and the toxicity of arsenic trioxide (ATO) was greatly reduced. *In vivo* pharmacokinetic experiments showed that plasma clearance rate of As_2_O_3_ liposomes was significantly reduced, and the *in vivo* circulation time was doubled compared with intravenous injection of As_2_O_3_ solution, which improved drug distribution in tumor tissues. Further research showed that the strong skeleton formed by arsenic and metal ions increased the stability of the drug, and the liposome lasted in the body for 6 months longer than the original.

#### 2.1.2 Protein nanomaterials

Protein nanomaterials is a kind of nano-drug loading system composed of drugs and animal, plant or recombinant proteins as carriers. Protein nanomaterials have the advantages of biodegradability, good biocompatibility, low immunogenicity, high drug-loading and stability, and can accumulate in tumor through EPR effect (Ding et al., 2020; Carrion et al., 2021). The types of protein nanomaterials commonly used in tumor therapy include albumin, whey protein, lipoprotein, zein, soy protein, recombinant ferritin and filamentin (Katouzian and Jafari, 2019).

Wamel’s (van Wamel et al., 2016) study showed that paclitaxel albumin nanoparticle (Abraxane) uses human serum albumin as a carrier, and through the natural transport pathway of albumin in the body, it can rapidly distribute and aggregate in tumor tissues, improve the efficacy of chemotherapy and reduce toxicity. Jayaprakasha et al. (2016) prepared whey protein-coated curcumin nanoparticles by solvent removal method. Compared with free curcumin, the uptake of colon cancer cells and prostate cancer cells increased significantly. The cytotoxicity and bioavailability of nano curcumin were significantly improved. Wu et al. (2013) prepared paclitaxel-fibroin nanoparticles (PTX-SF-NPs) in aqueous solution at room temperature for the treatment of local gastric cancer. The anti-tumor effect of PTX-SF-NPs was evaluated on the *in vitro* model of gastric cancer in nude mice, and the results showed that PTX-SF-NPs significantly enhanced the effect of delaying tumor growth and reducing tumor weight, with high safety. Sonekar et al. (2016) prepared folic acid conjugated curcumin-melololin nanoparticles by desolvation method, which can be taken orally and targeted for delivery to colon cancer cells, significantly improving the bioavailability and targeting of drugs.

#### 2.1.3 Polymeric nanomaterials

Polymeric nanoparticles are colloidal systems with a wide size range (10–1000 nm). Polymeric nanoparticles have high immunogenicity and stability, and can effectively encapsulate and display antigens. Polymeric nanoparticles can effectively protect drugs from *in vitro* and *in vivo* degradation, cross the blood-brain barrier, control drug release sites and improve drug targeting, and thus have a better therapeutic prospect in the targeted delivery of anti-tumor drugs (Elzoghby, 2017). In general, polymeric nanocarriers are more stable than liposomes. Common polymeric nanomedicine delivery carriers include polymeric micelles, dendritic macromolecules, polymeric nanogels, polymeric nanospheres, etc (Salari et al., 2022).

As is shown in Figure 3B, Galliani et al. (2019) developed a PLGA-based nanostructure, which can protect the structure of the enzymes from being destroyed and accurately deliver it to the cytoplasm. In the Figure 3C, Polyacaprolactone (PCL) nanoparticles loaded with Amp B were developed. The IC50 of the nanoformulations was significantly lower as compared to free Amp B, so it can greatly enhance the effect of Amp B. Wang et al. (2017) prepared gemcitabine (GEM) chitosan nanoparticles (FA-PEG-GEM-NPs) with surface modification of folic acid (FA) and polyethylene glycol (PEG). It was confirmed by cell experiments that FA-PEG-GEM-NPs had high selectivity and high toxicity to human non-small cell lung cancer A549 cells. Wu et al. (2018) designed a stimulus-responsive drug delivery system combining radiotherapy and chemotherapy. They modified L-cysteine (L-Cys) on the surface of polyamidoamine (PAMAM) polymer. On the one hand, the sulfhydryl group of L-Cys can act as a radiation protection agent by removing free radicals. On the other hand, disulfide bonds formed by sulfhydryl groups can be used to trigger the release of anti-cancer drugs. Low doses of gamma rays (5 Gy) trigger reactive oxygen species (ROS) production, which breaks disulfide bonds and releases anticancer drugs DOX. Gu et al. (Wang et al., 2018) designed a therapeutic nano hydrogel. When injected *in situ* into the tumor, the coated gemcitabine (GEM) and immune checkpoint inhibitor anti-PD-L1 antibody (aPDL1) were released in response, realizing the synergistic tumor suppression by chemotherapy and immunization. This gel can achieve ROS responsive degradation and programmed release of therapeutic drugs in ROS rich tumor microenvironments. The results showed that nano gel containing aPDL1-GEM significantly promoted immune-mediated tumor killing in tumor-bearing mice, thus it greatly prevented the recurrence of tumor after resection. Docetaxel (DTX) is a first-line chemotherapy drug for the treatment of metastatic breast cancer. Encapsulated in alendronate (AlN)-modified micelles, docetaxel can achieve sustained release and improve pharmacokinetics. DTX micelles can inhibit tumor growth and significantly prolong animal survival in a model of advanced disseminated breast cancer with bone metastases (Liu, 2019). Yan et al. Repoated a polymer-Based nanocarrier for Co-Delivery and combination of diverse therapies against cancers (Figure 3D).

### 2.2 Inorganic nanomaterials

Inorganic nanomaterials are nanocarriers synthesized by metallic and semi-metallic materials, which can be used for drug delivery. At present, inorganic nanomaterials used in tumor therapy mainly include metal nanomaterials, non-metallic nanomaterials, magnetic nanomaterials, etc, (Pei et al., 2023). Inorganic nanomaterials are easy to prepare and modify, which has become one of the important research directions of nanocarriers.

#### 2.2.1 Metallic nanomaterials

Metallic nanomaterials, which are synthesized based on metallic elements, not only have the function of targeted drug delivery of conventional carriers, but also enhance the ability of chemotherapy drugs to interfere with cell metabolism, inhibit proliferative cell signal transduction and induce cell apoptosis (Jin et al., 2018).

**FIGURE 4:**
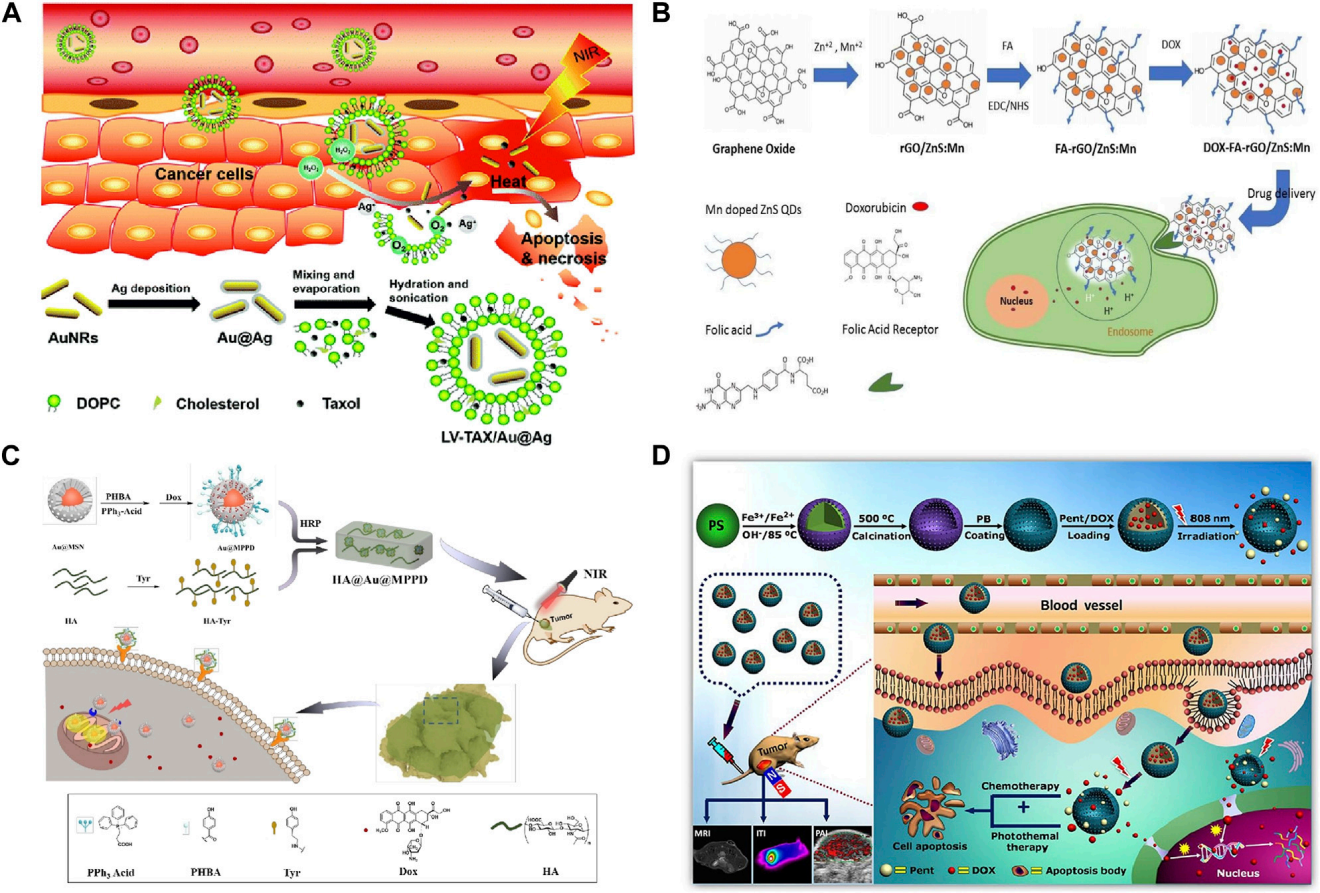
**(A)** Combination therapy of LV-TAX/Au@Ag (Wang K. et al., 2020a). **(B)** FA-rGO/ZnS:Mn as a drug delivery system to release DOX into the cancer cell nucleus (Diaz-Diestra et al., 2018). **(C)** FA-rGO/ZnS:Mn as a drug delivery system to release DOX into the cancer cell nucleus (Zhou et al., 2020). **(D)** Magnetic nanoparticles enhance drug targeting through magnetic fields (Estelrich et al., 2015; Gul et al., 2019).

In the Figure 4A, a hydrogen peroxide (H_2_O_2_)-triggered nanomaterial (LV-TAX/Au@Ag) was developed. When the nanomaterial reach the tumor tissues, it will release taxol and recovere the photothermal properties of AuNRs. With this combined chemo-photothermal therapy, tumor growth was significantly inhibited. Sun et al. (2015) prepared BioMOF ZnBTCA as a drug delivery vector to promote the uptake and release of anticancer drugs, which showed strong cytotoxicity to A2780cis (ovarian cancer cells) *in vitro*. ZnBTCA itself is not significantly cytotoxic to cancer cells, while drug@ZnBTCA will release the drug continuously and slowly to inhibit the cell growth of A2780cis. Compared with free drugs, drug@ZnBTCA reduces toxicity to normal cells. Tomuleasa et al. (2012) studied the therapeutic effects of different Au NPs loaded with adriamycin, cisplatin or capecitabine respectively on liver cancer, and the drug molecules were non-covalently combined with aspartic acid coated Au NPs. Compared with free drugs, Au NP-drug conjugations significantly improved the therapeutic efficacy of tumor cells, and were equally effective on drug-resistant cells. Rotello et al. (Zhu et al., 2008) demonstrated that Au NPs with surface charge and hydrophobicity are very conducive to cell uptake. Silver nanomaterials (Ag NPs) exhibit dose-dependent toxicity to tumor cells, killing tumor cells by inducing oxidative stress and DNA damage. TNBC tumor cells are sensitive to oxidative stress and DNA damage. Ag NPs have high selectivity for TNBC tumor cells, and have little damage to normal breast tissue cells, liver, kidney, and monocyte system cells (Simard et al., 2016). Curcumin and its derivatives are effective on TNBC tumor cells cultured *in vitro*, and it has been found that curcumin has greater damage to normal cells *in vivo* experiments. Li et al. (2017) found that in TNBC mouse model, the use of copper nanocomplex coating curcumin can improve its anticancer activity without significant adverse reactions (Li et al., 2017).

#### 2.2.2 Nonmetallic nanomaterials

At present, the most widely used nonmetallic nanomaterials mainly include carbon nanomaterials and silica nanomaterials. Mesoporous silica nanoparticles (MSN) have a larger specific surface area due to their unique mesoporous structure, and thus have better drug loading capacity.

In the Figure 4B; Diaz-Diestra et al. (2018) developed a nanocomposite (FA-rGO/ZnS:Mn). This nanocomposite can increase the drug loading rate and enhance the anti-tumor effect without increasing the toxicity to normal cells. Xiong et al. (2015) designed a novel MSN modified with two ligand (folic acid and dexamethasone), as a targeted delivery system for tumor nuclei. Folic acid modification can increase the targeting ability of MSN to tumor cells. Dexamethasone is a glucocorticoid, which can bind to the nuclear receptor of tumor cells, namely glucocorticoid receptor, and the formed dexamethasone-glucocorticoid receptor complex can be actively transported from cytoplasm to nucleus. By combining these two ligand to MSN, the novel delivery vector will target DOX into the tumor nucleus and increase its accumulation in the nucleus, thereby improving the inhibition of HeLa (human cervical cancer cells) and reducing the toxic and side effects on normal cells through receptor-mediated cell selectivity. Kumar et al. (2017) successfully prepared DOX-FA-MSNPs nanomaterials loaded with doxorubicin based on MSNs, and examined the uptake at the level of breast cancer cells by confocal microscopy and flow cytometry, and the results indicated that the uptake of nanomaterials in cell lines was high. Cytotoxic results showed that DOX-FA-MSNPs had higher cytotoxic effects on breast cancer cells. Khodadadei et al. (2017) loaded methotrexate (MTX) onto nitrogen-containing graphene quantum dots (GQDs) and found that its entry into tumor cells was slower than that of free MTX, but its effect time was longer and it had strong cytotoxicity. Liu et al. (2022) prepared nano-scale functional hydroxyapatite particles by reversibly combining hydroxyapatite with adriamycin. The nanoparticle is applied locally to the lesion area and can enter the lysosome of tumor cells. In acidic environment, the combination of hydroxyapatite and adriamycin is destroyed, and the released adriamycin accumulates in mitochondria. Further tests on osteosarcoma mice showed that local delivery of adriamycin via hydroxyapatite granules had a stronger tumor eradication effect than traditional application of adriamycin. Mesoporous silica is also a commonly used nanomaterial. A hyaluronic acid (HA) hydrogel covalently embedded with doxorubicin loaded and triphenylphosphine (TPP) modified core-shell gold mesoporous silica nanoparticles was developed by Zhou et al. (2020) This nanomaterial can accurately target tumor cells via CD44 and generate chemophotothermal synergistic cancer therapy (Figure 4C).

#### 2.2.3 Magnetic nanomaterials

Magnetic nanoparticles (MNPs) have magnetic properties and can be targeted to the target area by an external magnetic field. MNPs are generally magnetic composite materials composed of iron, nickel, cobalt and other metals and their oxides, which have been extensively studied in tumor targeted drug delivery. At present, magnetic nanocarriers used for tumor therapy mainly include core-shell structure nanoparticles (made from silica or polymeric micelles wrapped magnetic nuclei and drugs) (Oh and Park, 2011), magnetic liposomes (Song et al., 2020), and magnetic nanoparticles (transport modes are determined by particle size, morphology, surface charge, surface-to-coupled drugs or targeted molecules) (de Smet et al., 2011). Magnetic ferric oxide nanoparticles (MNP-Fe_3_O_4_) are magnetic nanomaterials approved by FDA for clinical application, which have attracted wide attention in the field of tumor targeted therapy (Zhao et al., 2018). In addition, multifunctional magnetic nanoagents (MMNs) is of great value for cancer precision therapy (Ding et al., 2021).

Figure 4D shows the process of a magnetic nanomaterial accurately delivering drugs to tumor tissues through magnetic fields, and killing tumors in coordination with photothermal effects. Wu et al. (2020) developed) a novel chitosan superparamagnetic ferric oxide nanocrystals (PECs), loaded with indocyanine green (ICG) fluorescent dye and irinotecan (IRT). By applying magnetic field, real-time fluorescence monitoring of the efficiency of magnetic targeted drug delivery to the tumour was realized. Centelles et al. (2018) designed imaged thermosensitive liposomes (iTSLs) for targeted Hycamtin delivery. iTSL accumulation at the tumor site can be detected by near infrared fluorescence imaging (NIRF), and its release at the tumor site can be detected by topotecan enhanced fluorescence. High intensity focused ultrasound (HIFU) can promote drug release from liposomes *in vivo*, induce subablative hyperthermia, change the permeability of tumor vessels and enhance the absorption of nanoparticles. Shao et al. (2016) designed a kind of magnetic Fe_3_O_4_/mesoporous silicon “nano bullet” particles. The magnetic Fe_3_O_4_ nanoparticles of “nano bullet” can provide magnetic targeting ability, and mesoporous silicon can adsorb chemotherapy drugs Dox. Therefore, the designed “nanobullets” can be targeted and enriched to the liver cancer area under the effect of magnetic targeting to realize the chemotherapy effect on tumors. Lu et al. (2018) constructed a PH-sensitive dual-targeting magnetic nanoparticle, and prepared graphene oxide (MGO) by deposition of Fe_3_O_3_ magnetic nanoparticles on graphene oxide (GO) by chemical coprecipitation. MGO- PEG-CET was obtained by modifying MGO with polyethylene glycol (PEG) and cetuximab (CET). EGFR is highly expressed on tumor cell surface, so MGO-PEG-CET can be used for dual targeted delivery of DOX.

## 3 Summary and outlook

Drug loading and targeted delivery of nanomaterials is one of the hot spots in biomedical research. Although many studies have demonstrated their advantages, most nanomaterials are still in the experimental stage and need to be improved in many aspects: 1) The biological toxicity of materials should be reduced as much as possible; 2) The pharmacokinetic characteristics, metabolism, distribution and cumulative effects *in vivo* need to be clarified. 3) How to improve the drug loading rate and the controllability of drug release of nanomaterials more effectively; 4) To improve the targeting of nanomaterials and reduce the toxicity to normal cells as much as possible. The real clinical use of nanomaterials requires more interdisciplinary and cross-field collaborative research. With the deepening of various researches, the application of nanomaterials will continue to make breakthrough progress.
